# Effects of Moxa Cone Moxibustion Therapy on Cognitive Function and Brain Metabolic Changes in MCI Patients: A Pilot ^1^H-MRS Study

**DOI:** 10.3389/fnagi.2022.773687

**Published:** 2022-05-26

**Authors:** Wei Mai, Aizhen Zhang, Qiang Liu, Liying Tang, Yichen Wei, Jiahui Su, Gaoxiong Duan, Jinlong Teng, Xiucheng Nong, Bihan Yu, Chong Li, Lijuan Shao, Demao Deng, Shangjie Chen, Lihua Zhao

**Affiliations:** ^1^Guangxi University of Chinese Medicine, Nanning, China; ^2^Department of Traditional Chinese Medicine, Guangxi Tumour Hospital, Nanning, China; ^3^Guangxi Medical College, Nanning, China; ^4^Xinghu Outpatient Department, The People’s Hospital of Guangxi Zhuang Autonomous Region, Nanning, China; ^5^Department of Radiology, The First Affiliated Hospital, Guangxi University of Chinese Medicine, Nanning, China; ^6^Department of Acupuncture, The First Affiliated Hospital, Guangxi University of Chinese Medicine, Nanning, China; ^7^Department of Radiology, The People’s Hospital of Guangxi Zhuang Autonomous Region, Nanning, China; ^8^Department of Rehabilitation, The Second Affiliated Hospital of Shenzhen University, Shenzhen, China

**Keywords:** moxa cone moxibustion, hydrogen proton magnetic resonance spectroscopy (1H-MRS), mild cognitive impairment (MCI), metabolites, cognitive function, hippocampus, posterior cingulate gyrus

## Abstract

**Objective:**

To explore the effect of moxa cone moxibustion on *N*-acetyl aspartate/total creatinine (NAA/tCr) and choline/total creatinine (Cho/tCr) in the bilateral hippocampus (HIP) and bilateral posterior cingulate gyrus (PCG) in patients with mild cognitive impairment (MCI) using hydrogen proton magnetic resonance spectroscopy (^1^H-MRS) and to provide imaging basis for moxa cone moxibustion treatment for MCI.

**Methods:**

One hundred eight patients with MCI were served as the MCI group, and 67 age-matched subjects were enrolled as the normal control group. The MCI group was randomized and allocated into acupoint group, drug group, and sham acupoint group, with 36 cases in each group. Some patients in each group withdrew. Finally, 25 cases were included in the acupoint group, 24 cases in the drug group, and 20 cases in the sham acupoint group. The drug group was treated with oral donepezil hydrochloride. The acupoint group and sham acupoint group received moxa cone moxibustion treatment. Mini-mental state exam (MMSE) and Montreal cognitive assessment (MoCA) scores were recorded before intervention, at the end of the first and the second months of intervention, and in the 5th month of follow-up. The NAA/tCr and Cho/tCr ratios in the HIP and PCG were bilaterally measured by ^1^H-MRS before and after intervention.

**Results:**

Before intervention, compared with the normal control group, the MMSE and MoCA scores, the Cho/tCr ratio in the right HIP, the NAA/tCr ratio in the bilateral HIP, and the NAA/tCr ratio in the left PCG in the three treatment groups decreased significantly (both *p* < 0.01), and the NAA/tCr ratio in the right PCG significantly reduced in the acupoint and drug groups (*p* < 0.05). After two months of treatment, compared with the normal control group, there were no differences in the MoCA scores, the NAA/tCr, and Cho/tCr ratios in the bilateral PCG and bilateral HIP in the three treatment groups (*p* > 0.05). However, the MMSE scores in the drug group decreased when compared with the acupoint group and normal control group (*p* < 0.05, *p* < 0.01). The scores of MMSE and MoCA in the acupoint group and sham acupoint group at all time points were better than those in the drug group, which were similar to those in the normal control group.

**Conclusion:**

Our findings suggest that moxibustion could improve the cognitive function of patients with MCI. The mechanism may be related to the improvement of abnormal brain metabolism in HIP and PCG.

## Introduction

Mild cognitive impairment (MCI) primarily presents with memory dysfunction and cognitive decline, and in some studies, there is a higher risk of progression to dementia compared to age-matched controls (Petersen et al., 2018). The morbidity of MCI in elderly people aged more than 60 years old ranged from 6.7 to 25.2%, and its occurrence is more common in men and increases with age and lower education (Suzuki et al., 2013; Langa and Levine, 2014; Cheng et al., 2017; Petersen et al., 2018). A systematic review and meta-analysis about preventing cognitive decline with non-pharmacological interventions (such as cognitive training, physical activity, and memory intervention) indicated that non-pharmacological therapy could effectively reduce the case of MCI or dementia among older people (Yao et al., 2020). However, there is currently no satisfactory treatment for MCI.

Hydrogen proton magnetic resonance spectroscopy (^1^H-MRS) can monitor the progression of disease and treatment response in neurodegenerative diseases (Oz et al., 2014).

^1^H-MRS, as a magnetic resonance (MR) technique that is non-invasive, with high sensitivity, and no radiation, has been widely used to evaluate changes in neurochemicals in specific brain regions in MCI and Alzheimer’s disease (AD) (Liu et al., 2021). The measurement of metabolite concentrations using ^1^H-MRS could provide new insights into the potential metabolic and microstructure changes of episodic memory impairment (Wong et al., 2020). *N*-acetyl aspartate (NAA) is present mainly in neurons, and a decrease in NAA predicts neuronal defects or dysfunction (Zhu et al., 2015) and has been found in different brain areas in variety of neurodegenerative diseases (Kuzniecky, 2004). The decrease of NAA mainly occurs at the time of disease progression. Creatine (Cr) mainly consists of creatine and creatine phosphate, which is associated with energy metabolism in the brain Chan et al., 1999; Gujar et al., 2005). In a lot of spectral research, Cr is commonly considered an internal reference for measuring other metabolites (Zhang et al., 2015; Fayed et al., 2017) except in high malignant tumors (Howe et al., 2003). Choline (Cho) reflects the impaired cholinergic neurons in AD and is considered to be a marker of cell turnover (Gujar et al., 2005). A prospective cohort study found that amnestic MCI (aMCI) would progress to AD with 74.1% sensitivity and 83.7% specificity when the NAA/Cr ratio in posteromedial bilateral parietal cortex was equal to or less than 1.43 (Modrego et al., 2011). A systematic review and meta-analysis of MRS in patients with MCI demonstrated that the reduction of NAA and NAA/Cr in the hippocampus (HIP) and posterior cingulate cortex (PCC) indicated the risk of progression to AD (Tumati et al., 2013). However, there were no significant differences in choline (Cho)/Cr ratio in the HIP and posterior cingulate (PC) area among AD, MCI, and normal subjects (Wang et al., 2009). Our previous study found that metabolite levels in HIP and posterior cingulate gyrus (PCG) in MCI subjects were correlated with their MMSE and MoCA scores and that MCI may occur when the NAA/tCr ratio if the bilateral HIP is less than 1.19 (Zhao et al., 2021). At the early stage, MCI provides a potential opportunity for therapeutic measures to prevent AD progression (Petersen et al., 2018). Therefore, it is very important to find easily available treatment measures to delay the progression of MCI to AD.

Donepezil is one of cholinesterase inhibitors (ChEIs), which is conducive to cognitive function and slows down the progression of MCI to AD (Edmonds et al., 2017). Neuroimaging research demonstrated that donepezil could slow down the rate of HIP atrophy and could have neuroprotective effects on AD (Hashimoto et al., 2005), while rivastigmine could reverse decreases in NAA/Cr ratio in AD (Modrego et al., 2006). However, a randomized trial with MRS showed that more patients worsened after donepezil or memantine treatment (Modrego et al., 2010). In China, donepezil was used to treat amnestic mild cognitive impairment (aMCI), which showed improvement in clinical symptoms and neuropsychological tests (Miao et al., 2011; Zhao and Zhao, 2012). Although ChEIs have a slight efficacy in the treatment of MCI, there are many safety issues, such as dizziness, diarrhea, insomnia, and so on, and the incidence of discontinuation due to adverse events is high (Matsunaga et al., 2019).

Moxibustion is a safe treatment, and lots of clinical reports have demonstrated that moxibustion is effective with few adverse events (Xu et al., 2014). Moxibustion is a therapy that uses the heat generated by burning dried wormwood to stimulate one or more relevant acupoints. Research has proven that Baihui (DU20), Guanyuan (RN4), Zusanli (ST36), and Xuanzhong (GB39) acupoints, normal control (NC) have positive effects on cognitive function, especially in MCI and AD (Liu et al., 2011; Gu et al., 2014; Wang et al., 2020; Kim et al., 2021). In addition, our previous study also demonstrated that moxibustion at Baihui (DU 20), Guanyuan (RN 4), Zusanli (ST 36), and Xuanzhong (GB 39) acupoints could improve the cognitive function of MCI (Zhao et al., 2019). Moxibustion could reduce neuronal edema in the HIP and has a beneficial effect for preventing the progression of AD (Du et al., 2013; Zhou et al., 2014). In China, a series of clinical trials and animal studies have been conducted to explore the benefits and mechanisms of moxibustion in the prevention and treatment of MCI (Choe et al., 2018; Zhao et al., 2019; Wang et al., 2020). Although moxibustion has a long history of use and clinical and experimental support, the mechanism of moxibustion treatment for MCI remains unclear.

Here we used ^1^H-MRS to investigate the effects of moxa cone moxibustion on brain metabolisms in HIP and PCG in patients with MCI. We hypothesized that moxa cone moxibustion has a regulatory effect on the abnormal brain metabolisms in HIP and PCG.

## Methods

The study protocol was designed and implemented according to the principles of the Declaration of Helsinki. All the subjects (or their legal representative) signed the informed consent. The Clinical Trial Registration Number of this study is ChiCTR-IPR-16009144 and has been registered at http://www.chictr.org.cn. The protocol of this study was approved by the Ethics Committee of The First Affiliated Hospital of Guangxi University of Chinese Medicine [Lot number: (2016) 009].

### Study Design, Randomization, and Blinding

This study was a randomized, drug-controlled, parallel group trial to explore the efficacy and safety of moxa cone moxibustion and donepezil on the treatment of patients with MCI from January 2014 to December 2018. All MCI subjects came from the older residents with complaints of cognitive decline. The survey was conducted in the First Affiliated Hospital of Guangxi University of Chinese Medicine. All patients with MCI who met the inclusion criteria were randomly allocated into drug group, acupoint group, and sham acupoint group by computer-generated random numbers (random list generated by SPSS 22.0), and the random number table was sealed in a special envelope. The course of this study included pre-treatment (baseline) period, 2-month treatment period, and 3-month follow-up period. MMSE and MoCA scale assessments for all participants were performed before treatment, at the end of the first and the second months of treatment, and in the 5th month of follow-up. Cho/tCt and NAA/tCr ratios in the PCG and HIP of patients with MCI were detected by ^1^H-MRS before treatment and at the end of the second month of treatment.

Because of the nature of the intervention, physicians could not be blinded. The acupuncturists should be aware of the group intervention protocol owing to the manipulation. Data collection was performed by two blinded assessors. Data analysis was also performed blinded. All patients were treated as equally as possible except for the differences in treatment methods among the three groups. During the intervention, data collectors and acupuncturists would visit patients at different times to prevent them from exchanging information with each other.

### Participants

One hundred eight patients with MCI served as the MCI group and 67 age-matched subjects served as the normal control group. The subjects who took part in this study were recruited from January 2014 to December 2018 in the First Affiliated Hospital of Guangxi University of Chinese Medicine. The MCI group was randomized and allocated into acupoint group, drug group, and sham acupoint group, with 36 cases in each group. Some patients in each group withdrew. Finally, 25 cases were included in the acupoint group, 24 cases were included in the drug group, and 20 cases were included in the sham acupoint group.

The inclusion criteria were as follows: (1) educated elderly aged 55–82, (2) patients with MCI with memory impairment as their chief complaint, an abnormal Montreal Cognitive Assessment (MoCA) score (Petersen, 2004; Petersen and Morris, 2005; Lu et al., 2011), and met the diagnostic criteria for MCI in the 2006 Chinese guidelines for the diagnosis and treatment of dementia (Tian, 2012). All patients with MCI who participated in this study had aMCI. (3) In the age-matched normal control group, there was no complaint of cognitive dysfunction, and the Mini-Mental Status Examination (MMSE) (Chinese version) score was more than 27 (Zhang, 2003), with normal overall cognitive function and no other brain injuries such as cerebral infarction.

The exclusion criteria were as follows: (1) clinical stroke and acute cardiovascular disease; (2) patients diagnosed with ataxia or with a history of ataxia; (3) patients diagnosed with malignant neoplastic diseases; and (4) patients with a previous history of excessive drinking, smoke, or substance abuse, and taking other medications that may lead to changes in cognitive function.

### Interventions

#### Acupoint Group

**FIGURE 1:**
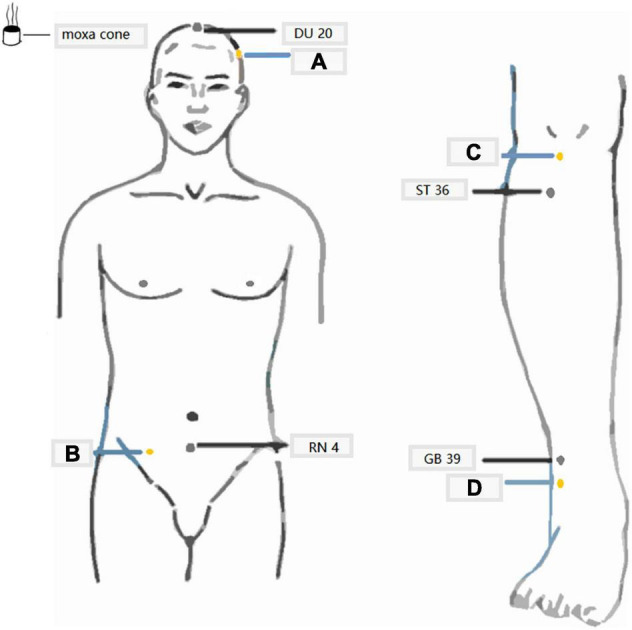
Schematic diagram of moxibustion treatment. Locations of selected acupoints and sham acupoints. The acupoints were Baihui (DU 20), Guanyuan (RN 4), Zusanli (ST 36, bilateral), and Xuanzhong (GB 39, bilateral). The sham acupoints were **(A–C)** (bilateral), and **(D)** (bilateral).

Baihui (DU20), Guanyuan (RN4), Zusanli (ST36, bilateral) and Xuanzhong (GB39, bilateral) (Shi, 2007; Figure 1). Sham acupoint group: (A) 3.33 cm above the Shuaigu (GB 8) on the left side of the head, (B) 10 cm on the right side of the RN 4, (C) the depression below the bilateral midpoint of the patellar tips and (D) 3.33 cm on the bilateral lateral malleolus tips (Figure 1).

#### Manipulation

Vaseline was applied to the acupoints to protect the patient’s skin and facilitate the fixation of moxa cone (size, 12 mm × 15 mm; Nanyang lvying moxa grass *Biological Products Co., Ltd.*). Three moxa-cones were burnt on each acupoint or sham acupoint in each treatment. Each acupoint or sham acupoint lasts approximately 3–5 min for each time. The acupoint group and sham acupoint group received treatment every other day, 15 times for one course, and rested for 2 days to continue the next course, for a total of two courses of intervention.

#### Drug Group

Five milligrams of oral donepezil hydrochloride [Produced by Weicai (China) Pharmaceutical Co., Ltd.: H20050978, batch number: 131224a, 130820a, 1506011, 1610044, 5 mg × 7 tablets] was prescribed to be ingested once every night for 30 days in one course, for a total of two courses of intervention.

### Proton MRI and Spectroscopy

The 3.0-Tesla Verio superconducting MRI system (Siemens, Munich, Germany) was applied to generate high-resolution T1-weighted images and multi-voxel ^1^H-MRS scans of the MCI and NC groups. The high-resolution T1-weighted images were used to localize the bilateral HIP and PCG. The parameters of high-resolution T1-weighted images were as follows: repetition time (TR)/the echo time (TE) = 1,900 ms/2.22 ms; field of view = 250 mm × 250 mm; flip angel = 9°; matrix size = 256 × 256; slice thickness = 1 mm for 176 slices; and bandwidth = 200 Hz. A point resolved spectroscopy-chemical shift imaging (PRESS-CSI) sequence was applied to ^1^H-MRS scanning, and the parameters of the ^1^H-MRS scans were as follows: TR/TE = 1,700/135 ms; bandwidth = 1,200 Hz; field of view (FOV) = 160 mm × 160 mm; flip angle = 90°; and matrix size = 16 × 16. The volume of interest (VOI) was 80 mm × 66 mm × 15 mm for bilateral HIP and 50 mm × 50 mm × 15 mm for bilateral PCG. The voxel size was 10.0 mm × 10.0 mm × 15.0 mm for both HIP and PCG. The HIP VOI size was designed to match the bilateral HIP, which was located in the skull base, to avoid interference from other factors, i.e., the skull or cerebrospinal fluid. An automatic pre-scanning program was applied to adjust the voxel gain, to receive/transmit, and to semi-automatically shim weak water suppression with full width at half maximum (FWHM) of less than 25 Hz and water suppression level of greater than 95%. After the scan, a sequence of postprocessing steps were employed to get the MRS data, including the water reference processing, filter, zero-filling, Fourier transformation, frequency shift correction, baseline correction, phase correction, and curve fitting. The chemical shifts of metabolites were 2.02 ppm for NAA, 3.03 ppm for total Cr (tCr), and 3.22 ppm for Cho. tCr was considered as internal parameter, the NAA/tCr and Cho/tCr ratios were calculated. The MoCA (Beijing version) and MMSE (Chinese version) were applied by the same clinician before the MRS detection in the patients with MCI and normal controls.

### Cognitive Function Assessment

The MMSE and MoCA scales were applied to assess the cognitive function of the participants. (1) MMSE (Chinese version) (Zhang, 2003) contains several tasks to assess multiple cognitive domains, including memory, orientation test, capacity of calculation, attention, language (naming, auditory comprehension, repetition, reading, and writing), and visual special ability. The total score of MMSE is 30, and the lowest score is 0. The lower the scores, the more serious the damage. The (2) MoCA (Beijing version) tests eight cognitive domains including attention, visuospatial ability, executive function, instant recall, delayed recall, abstraction, language, calculation, and orientation. MoCA score ranges from 0 to 30. The higher the scores, the better the cognitive function. If years of education were less than 12, the total measured score was the test score plus 1 point. All assessments were administered and graded by professionals trained in neuropsychological testing.

### Statistical Analysis

Statistical Package for the Social Sciences (SPSS) 22.0 software was applied to analysis the data. The enumeration data were analyzed with χ^2^ test. The measurement data that meet the normal distribution use the mean ± standard deviation (mean ± SD) statistical description and one-way ANOVA were used for comparison between groups. The measurement data of skewed distribution were described by median (P50) and compared between groups by rank sum test. Repeated measures ANOVA was used to check whether there were time differences in MMSE and MoCA scores among the groups. Greenhouse-Geisser was used to correct for dissatisfaction with the Mauchly’s Test of Sphericity. The difference was statistically significant at the level of *p* < 0.05.

## Results

### Participants and Demographic

**FIGURE 2:**
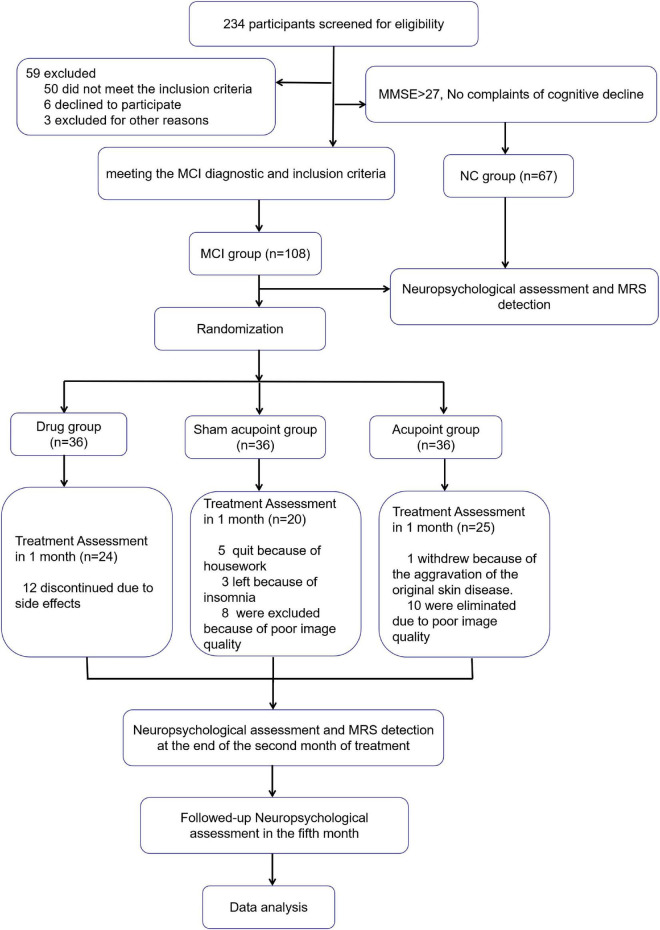
Flowchart of the study. MMSE, Mini-Mental State Examination; MoCA, Montreal Cognitive Assessment; MCI, Mild cognitive impairment; NC, normal control.

Figure 2 shows the details of the data collection process. From January 2014 to December 2018, 234 subjects participated in screening for eligibility. Fifty individuals were excluded for not meeting the inclusion criteria and 67 age-matched normal individuals were included as normal control group. A total of 108 participants in MCI group were randomly enrolled and allocated into the acupoint group (*n* = 36), sham acupoint group (*n* = 36), and drug group (*n* = 36). In the drug group, 12 patients withdrew from the experiment because of the adverse events of donepezil, such as insomnia and gastrointestinal symptoms. In the sham acupoint group, five patients stopped treatment due to housework, three patients withdrew because of insomnia, and eight patients were eliminated because of poor image quality. In the acupoint group, one patient failed to continue with the procedure due to exacerbation of a primary skin disease and 10 patients were eliminated due to poor image quality.

There were no significant differences in education levels, age, and gender among the four groups. There were also no significant differences in the course of disease among the drug group, sham acupoint group, and acupoint group (Table 1).

**TABLE 1:** Demographic data.

Variable	Acupoint group (*n* = 25)	Drug group (*n* = 24)	Sham acupoint group (*n* = 20)	Normal control group (*n* = 67)	χ^2^/*F*/*Z*-value	*P*-value
**Gender**						
Man (%)	9 (36%)	7 (29.2%)	4 (20%)	25 (37.3%)		
Female (%)	16 (64%)	17 (70.8%)	16 (80%)	42 (62.7%)	2.350	0.503
Age (years)	63.52 ± 6.20	66.00 ± 6.42	64.25 ± 7.50	64.76 ± 5.73	0.686	0.562
Education level (years)	11.36 ± 2.00	10.13 ± 2.82	10.85 ± 2.94	11.85 ± 3.04	2.410	0.070
Course of disease (months)	24.00 (19.50, 36.00)	28.00 (24.00, 45.00)	24.00 (24.00, 45.0)	/	0.877	0.645

*Data represent number, mean ± SD were analyzed by one-way ANOVA. Median P50 (P25, P75) were analyzed by rank sum test.*

### Outcome Measures Before Treatment

In comparison with the normal control group, the MMSE and MoCA scores in the three treatment groups significantly declined (*p* < 0.01). In the three treatment groups, the changes of metabolites in the bilateral HIP and bilateral PCG were compared with those in the normal control group, specifically, the NAA/tCr ratio in the bilateral HIP and the Cho/tCr ratio in the right HIP in the acupoint group. Meanwhile, sham acupoint group and drug group significantly reduced (*p* < 0.01). There were no significant differences in the Cho/tCr ratio in the left HIP between the three treatment groups and the normal control group (*p* > *0.05*). The NAA/tCr ratio in the left PCG in the three treatment groups and the NAA/tCr ratio in the right PCG in the acupoint group and the drug group was significantly reduced (*p* < 0.01, *p* < 0.05). There were no significant differences in the Cho/tCr ratio in the bilateral PCG between the three treatment groups and the normal control group (*p* > 0.05, Table 2).

**TABLE 2:** Cognitive scores and magnetic resonance spectroscopy (MRS) results before treatment.

Variable	Acupoint group	Drug group	Sham acupoint group	Normal control group	*F*-value	*P*-value
MMSE	25.96 ± 0.98**	25.54 ± 1.22**	26.15 ± 0.93**	29.13 ± 0.76	145.01	<0.001
MoCA	21.36 ± 2.68**	21.58 ± 2.96**	21.95 ± 2.72**	26.03 ± 2.01	37.677	<0.001
HIP.L NAA/tCr	1.05 ± 0.19**	1.10 ± 0.21**	1.17 ± 0.22**	1.38 ± 0.23	18.893	<0.001
HIP.L Cho/tCr	0.96 ± 0.17	0.94 ± 0.24	0.98 ± 0.28	1.00 ± 0.18	0.533	0.661
HIP.R NAA/tCr	1.05 ± 0.14**	1.07 ± 0.17**	1.16 ± 0.14**	1.36 ± 0.25	20.365	<0.001
HIP.R Cho/tCr	0.89 ± 0.19**	0.90 ± 0.18**	0.91 ± 0.20**	1.04 ± 0.19	7.042	<0.001
PCG.L NAA/tCr	1.81 ± 0.17**	1.85 ± 0.27**	1.95 ± 0.20*^#^	2.07 ± 0.21	11.453	<0.001
PCG.L Cho/tCr	1.06 ± 0.32	0.96 ± 0.14	1.06 ± 0.24	1.07 ± 0.18	1.566	0.201
PCG.R NAA/tCr	1.77 ± 0.20**	1.82 ± 0.30**	1.93 ± 0.21^#^	2.00 ± 0.24	7.399	<0.001
PCG.R Cho/tCr	0.96 ± 0.14	1.02 ± 0.19	1.09 ± 0.24	1.07 ± 0.22	2.162	0.095

MMSE, Mini-Mental State Examination; MoCA, Montreal Cognitive Assessment; L, Left, R, Right; HIP, hippocampus; PCG, posterior cingulate gyrus; NAA, N-acetyl aspartate; Cho, choline; tCr, total creatine; compared with the normal control group, **p < 0.01, *p < 0.05; compared with the acupoint group, ^#^p < 0.05.

### Outcome Measures After Treatment

After 2 months of treatment, there were no significant differences in the MMSE scores and MoCA scores among the acupoint group, sham acupoint group, and the normal control group (*p* > 0.05). However, the MMSE scores in the drug group decreased when compared with the acupoint group and normal control group (*p* < 0.05, *p* < 0.01). There were no significant differences in the NAA/tCr and Cho/tCr ratios in the bilateral HIP, and the NAA/tCr and Cho/tCr ratios in the bilateral PCG between the three treatment groups and the normal control group (*p* > 0.05, Table 3).

**TABLE 3:** Cognitive scores and MRS results after treatment.

Variable	Acupoint group	Drug group	Sham acupoint group	Normal control group	*F*-value	*P*-value
MMSE	29.00 ± 1.32	28.04 ± 2.35**^#^	28.50 ± 1.76	29.13 ± 0.76	3.976	0.009
MoCA	26.16 ± 3.17	24.92 ± 3.20	26.80 ± 1.96	26.03 ± 2.01	2.239	0.087
HIP.L NAA/tCr	1.40 ± 0.21	1.38 ± 0.21	1.29 ± 0.21	1.38 ± 0.23	1.145	0.333
HIP.L Cho/tCr	1.03 ± 0.19	1.06 ± 0.18	1.03 ± 0.23	1.00 ± 0.18	0.750	0.524
HIP.R NAA/tCr	1.41 ± 0.22	1.37 ± 0.23	1.26 ± 0.17	1.36 ± 0.25	1.589	0.195
HIP.R Cho/tCr	1.10 ± 0.28	1.09 ± 0.24	0.99 ± 0.18	1.04 ± 0.19	1.169	0.324
PCG.L NAA/tCr	2.08 ± 0.25	2.16 ± 0.32	1.98 ± 0.20	2.07 ± 0.21	2.063	0.108
PCG.L Cho/tCr	1.14 ± 0.22	1.13 ± 0.21	1.11 ± 0.19	1.07 ± 0.18	1.232	0.301
PCG.R NAA/tCr	2.09 ± 0.25	2.09 ± 0.27	1.96 ± 0.22	2.00 ± 0.24	1.757	0.159
PCG.R Cho/tCr	1.18 ± 0.23	1.11 ± 0.20	1.12 ± 0.23	1.07 ± 0.22	1.708	0.169

MMSE, Mini-Mental State Examination; MoCA, Montreal Cognitive Assessment; L, Left; R, Right; HIP, hippocampus; PCG, posterior cingulate gyrus; NAA, N-acetyl aspartate; Cho, choline; tCr, total creatine; compared with the normal control group, **p < 0.01; compared with the acupoint group, ^#^p < 0.05.

### Mini-Mental State Examination Scores

**FIGURE 3:**
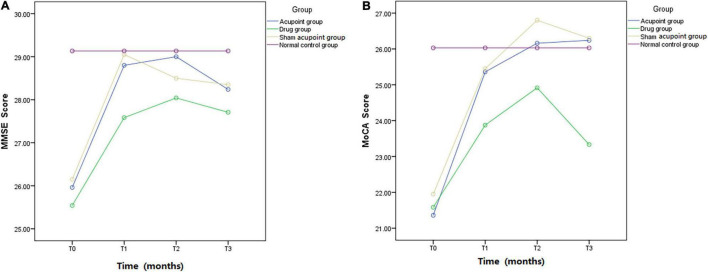
Mini-mental state examination and Montreal Cognitive Assessment. MMSE and MoCA scores in the acupoint group, drug group, sham acupoint group, and the normal control group.

The MMSE scores at all time points in the four groups are shown in Table 4. The results of repeated measures analysis of MMSE scores suggested a significant time effect (*p* < 0.0001) and group effect (*p* < 0.0001) and detected a time × group interaction (*p* < 0.0001). At the end of the second month of treatment, the MMSE scores in the acupoint group and sham acupoint group were better than those in the drug group. During the follow-up at the 5th month, it was found that the MMSE scores of the acupoint and sham acupoint groups were still high (Figure 3A).

**TABLE 4:** Mini-mental state examination (MMSE) scores in four groups at different time points.

Group	T0	T1	T2	T3
	Baseline	First month	Second month	Fifth month
Acupoint group	25.96 ± 0.98	28.80 ± 1.29	29.00 ± 1.32	28.24 ± 2.18
Drug group	25.54 ± 1.22	27.58 ± 2.45	28.04 ± 2.35	27.71 ± 2.80
Sham acupoint group	26.15 ± 0.93	29.05 ± 1.19	28.50 ± 1.76	28.35 ± 0.67
Normal control group	29.13 ± 0.76	29.13 ± 0.76	29.13 ± 0.76	29.13 ± 0.76

*T0, pretreatment; T1, at the end of the first month of treatment; T2, at the end of the second month of treatment; T3, in the fifth month of follow-up.*

### Montreal Cognitive Assessment Scores

The MoCA scores at all time points in the four groups are listed in Table 5. A time effect (*p* < 0.0001), time × group interaction (*p* < 0.0001), and group effect (*p* < 0.0001) were detected in the analysis of MoCA scores using a repeated measures approach. The MoCA scores in the acupoint and sham acupoint groups remained at a high level in the 5th month of follow-up, while the drug group showed a downward trend (Figure 3B).

**TABLE 5:** Montreal cognitive assessment (MoCA) scores in four groups at different time points.

Group	T0	T1	T2	T3
	Baseline	First month	Second month	Fifth month
Acupoint group	21.36 ± 2.68	25.36 ± 3.24	26.16 ± 3.17	26.24 ± 3.39
Drug group	21.58 ± 2.96	23.88 ± 3.08	24.92 ± 3.20	23.33 ± 3.95
Sham acupoint group	21.95 ± 2.72	25.45 ± 1.85	26.80 ± 1.96	26.30 ± 1.69
Normal control group	26.03 ± 2.01	26.03 ± 2.01	26.03 ± 2.01	26.03 ± 2.01

*T0, pretreatment; T1, at the end of the first month of treatment; T2, at the end of the second month of treatment; T3, in the fifth month of follow-up.*

### Cases

**FIGURE 4:**
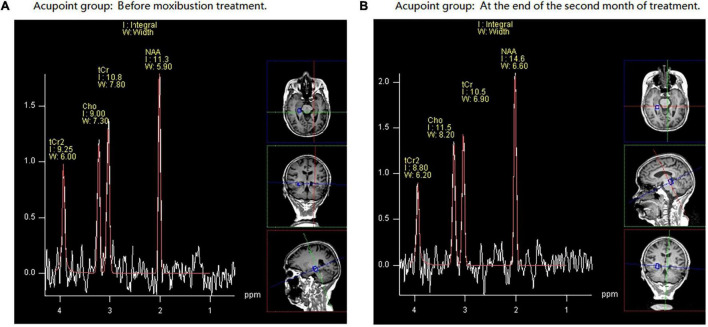
An example of hydrogen proton magnetic resonance spectroscopy (^1^H-MRS) data obtained in a 74-year-old female volunteer with mild cognitive impairment (MCI) from acupoint group. NAA, *N*-acetyl aspartate; Cho, choline; tCr, total creatine. Part **(A)** demonstrates that before moxibustion treatment, in the right hippocampus (HIP), the *N*-acetyl aspartate/total creatinine (NAA/tCr) ratio is 1.05 and choline/total creatinine (Cho/tCr) ratio is 0.84. Part **(B)** shows that at the end of the second month of treatment, in the right HIP, the NAA/tCr ratio is 1.40 and Cho/tCr ratio is 1.10.

Figures 4A,B demonstrate a case from the acupoint group. It reports the changes of the Cho/tCr and NAA/tCr ratios in the right HIP before (A) and after (B) moxa cone moxibustion treatment.

## Discussion

In the present study, we performed ^1^H-MRS to detect the effects of moxa cone moxibustion and donepezil on the brain metabolisms in HIP and PCG in patients with MCI and normal controls. We found that the NAA/tCr ratios in the bilateral HIP, bilateral PCG, and the Cho/tCr ratio in the right HIP in patients with MCI were different from those of the normal subjects. After 2 months of treatment, patients with MCI in the acupoint, sham acupoint, and donepezil groups showed increases in the NAA/tCr ratios in the bilateral HIP, bilateral PCG, and the Cho/tCr ratio in the right HIP. In addition, there were no significant differences among the three treatment groups. However, the MMSE scores in the acupoint group were higher than those in the drug group. In the 5th month of follow-up, we observed that compared with the drug group, the MMSE and MoCA scores of acupoint and sham acupoint groups maintained an elevated level.

Hydrogen proton magnetic resonance spectroscopy of the HIP in MCI and AD subjects showed that decreased NAA and NAA/Cr ratio may forecast the progression of MCI to AD in the future (Targosz-Gajniak et al., 2013; Gao and Barker, 2014). The levels of NAA are related to the cognitive decline and could be used to discriminate between healthy controls, MCI, and AD (Liu et al., 2021). In the current study, we found decreased NAA/tCr ratios in the bilateral HIP in patients with MCI of acupoint, sham acupoint, and drug groups than those in the normal controls. The HIP is an important hub of the neural network for learning and memory, and any pathological changes in this region may lead to memory disorder (Squire and Wixted, 2011; Ezzati et al., 2016). The reduction of NAA/Cr ratio in the right HIP indicated that it might have early changes in the right HIP region in patients with mild memory impairment (MMI) (Caserta et al., 2008). The decrease of Cho level in HIP in MCI may reflect a compensatory mechanism of the increased Choline Acetyltransferase (ChAT) activity necessary for the decrease of cholinergic input (Tumati et al., 2013). In the present study, in patients with MCI of acupoint, sham acupoint, and drug groups, the levels of Cho/tCr in the right HIP were significantly decreased. However, in comparison with the normal controls, there were no reduction in Cho/tCr in the left HIP among the three treatment groups. We speculate that this may be related to the asymmetry changes in the HIP. Previous findings also reported that a left-less-than-right pattern of the HIP volume was found in MCI, and the right HIP was more atrophic than the left (Shi et al., 2009; Minkova et al., 2017). The decrease of Cho/tCr in the right HIP are consistent with a previous study (Zhao et al., 2021). The left-right asymmetry of Cho/tCr in HIP found in this study may be associated with the right-handedness of patients with MCI. The HIP shows a variety of structural, neurochemical, molecular, and cellular changes during MCI, supporting its role as a center of neuroplasticity remodeling in the medial temporal lobe of the brain. On the other hand, the HIP neuroplasticity pathways provide a compelling basis for therapeutic intervention (Mufson et al., 2015). After 2 months of treatment, we found that the NAA/tCr in the bilateral HIP and the Cho/tCr in the right HIP in the three treatment groups increased and that there were no differences in the NAA/tCr and Cho/tCr ratios in the bilateral HIP among the three treatment groups, which indicated that the regulatory effect of moxibustion on abnormal metabolites in HIP was comparable with that of donepezil. Previous research reported that moxibustion could enhance the resting state functional connectivity between bilateral HIP and other brain regions, such as the precuneus and inferior parietal lobe (Bao et al., 2017). The regulation of moxibustion on brain function is not in a single brain region, but in the network of multiple brain regions, and the nervous system participates in the heat sensitivity induced by moxibustion (Xie et al., 2013; Chen et al., 2019). Interestingly, in this study, we observed that the improvement of MMSE scores in the acupoint group was better than in the drug group. Besides, the sham acupoint group also showed improvement in MMSE and MoCA scores, and the long-term effect of the acupoint and sham acupoint groups in improving the scores of MMSE and MoCA was better. Thermal stimulation is the crucial factor affecting the curative effect of moxibustion therapy (Pach et al., 2009; Yi, 2009). The improvement of MMSE and MoCA scores in sham acupoint group may be related to the heat conduction and radiation produced by moxibustion intervention. However, the special roles of sham acupoints in patients with MCI still require further investigation. In summary, our research suggests that patients with MCI display decreased NAA/tCr in the bilateral HIP and the Cho/tCr in the right HIP, which could be improved by moxibustion together with cognitive function improvement.

In this study, we observed that NAA/tCr ratios in the bilateral PCG in MCI subjects were lower than those in the normal controls. A meta-analysis also reported a significant reduction in NAA/Cr ratio in the PC in patients with MCI (Tumati et al., 2013). Neuroimaging studies have identified the PCG as a cortical region that is affected early in the onset of AD (Scheff et al., 2015). Metabolic abnormalities can be observed in neurodegenerative diseases in the HIP and PCC, which are known for their participation in MCI (Oeltzschner et al., 2019; Zhao et al., 2021). Reduced metabolism in the PCC is an early feature of AD and is often present before a definitive clinical diagnosis (Minoshima et al., 1997; Johnson et al., 1998). The reduction of NAA/Cr ratio in PCG of AD and aMCI is correlated with cognitive dysfunction and reflects neuronal loss (Lim et al., 2012). In MCI, lower levels of neuronal integrity marker NAA to Cr ratio in the PCG on MRS is related to a higher risk of progression to AD (Kantarci et al., 2007). Our findings in the current study are in accordance with a previous research which states that the decreased NAA/Cr level is an indicator of early neurodegeneration in the PCG of patients with MCI who progressed to AD (Zhang et al., 2015). Therefore, we speculate that the decreased NAA/tCr ratios in the bilateral PCG reflect the neuronal loss, which might lead to the cognitive dysfunction in patients with MCI. However, findings with respect to the Cho levels and Cho/Cr ratio in the PCG are inconsistent. For example, Wang et al. (2009) observed that in PC area, there were no significant differences in the Cho/Cr ratios among the patients with MCI, AD, and normal subjects. Consistent with the previous research, in this study, we also found that there were no differences in Cho/tCr ratios in the bilateral PCG between patients with MCI and normal controls. While Su et al. (2016) found that Cho/Cr levels reduced in PC in AD subjects. By contrast, Kantarci et al. (2000) found that the Cho/Cr ratio in the PC VOI increased in patients with AD. After 2 months of treatment, NAA/tCr ratios in the bilateral PCG in the acupoint and drug groups significantly increased, and NAA/tCr ratios in the bilateral PCG in sham acupoint group also slightly increased. After 2 months of treatment, NAA/tCr ratios in the bilateral PCG in the acupoint and drug groups significantly increased, and NAA/tCr ratios in the bilateral PCG in sham acupoint group slightly increased. During moxibustion treatment, the acupoints and sham acupoints of patients were always kept warm. The warm stimulation of moxibustion makes the patient feel comfortable and pay his or her attention to the therapeutic acupoints or sham acupoints. The local thermal effect and spectral radiation produced by moxibustion may be the mechanism for treating MCI (Yang and Hu, 2009). In addition, we also observed that the effect of moxibustion in regulating the abnormal level of NAA/tCr in the PCG was comparable with that of donepezil. Though moxibustion treatment may lead to burns, it could be avoided if it is operated carefully. In addition, it is painless, easy to operate, with few adverse reactions. According to the findings of this study, we speculate that the improvement of NAA/tCr ratios in the bilateral PCG may demonstrate the improved cognitive function in patients with MCI after moxibustion treatment.

### Limitations

There were some limitations in our study. Particularly, a small sample of patients with MCI was included, clinical follow-up at 3 months after treatment only assessed the patients with MMSE and MoCA scales, and metabolites in HIP and PCG were not detected again, which could not fully understand the changes of cerebral metabolites in patients with MCI at 3 months after the end of moxibustion treatment. Multimodal functional MRI, such as blood oxygenation level dependent (BOLD) imaging and diffusion tension imaging (DTI), should be applied in future studies to comprehensively assess the efficacy of moxibustion for preventing MCI.

## Conclusion

We investigated the NAA/tCr and Cho/tCr ratios in the bilateral HIP and bilateral PCG in patients with MCI compared with normal controls and explored the effect of moxa cone moxibustion treatment on the NAA/tCr and Cho/tCr ratios in the bilateral HIP and bilateral PCG in MCI. Patients with MCI showed reductions in NAA/tCr in the bilateral HIP, bilateral PCG, and Cho/tCr in the right HIP, which might result in cognitive dysfunction. Our study suggests that regulations of NAA/tCr in the bilateral HIP, bilateral PCG, and Cho/tCr in the right HIP may be the major pattern of brain response to moxibustion treatment for MCI.

## Data Availability Statement

The original contributions presented in the study are included in the article/supplementary material, further inquiries can be directed to the corresponding authors.

## Ethics Statement

The studies involving human participants were reviewed and approved by The First Affiliated Hospital of Guangxi University of Chinese Medicine [Lot number: (2016)009]. The patients/participants provided their written informed consent to participate in this study.

## Author Contributions

LZ and WM designed the trial, provided the theory of this work, analyzed and interpreted the data, and wrote and revised the manuscript. WM, AZ, QL, LT, JT, JS, XN, BY, and LS were mainly responsible for collecting the data, acquiring images, and performing the experiments. SC, DD, CL, GD, and YW collected image data. All authors read and approved the final manuscript.

## Conflict of Interest

The authors declare that the research was conducted in the absence of any commercial or financial relationships that could be construed as a potential conflict of interest.

## Publisher’s Note

All claims expressed in this article are solely those of the authors and do not necessarily represent those of their affiliated organizations, or those of the publisher, the editors and the reviewers. Any product that may be evaluated in this article, or claim that may be made by its manufacturer, is not guaranteed or endorsed by the publisher.
